# LEAD-YOLO: A Lightweight and Accurate Network for Small Object Detection in Autonomous Driving

**DOI:** 10.3390/s25154800

**Published:** 2025-08-04

**Authors:** Yunchuan Yang, Shubin Yang, Qiqing Chan

**Affiliations:** School of Electrical and Information Engineering, Wuhan Institute of Technology, Wuhan 430205, China; 15936194700@163.com (Y.Y.); xiaochan1207@163.com (Q.C.)

**Keywords:** autonomous driving, object detection, YOLOv11n, small object, lightweight

## Abstract

The accurate detection of small objects remains a critical challenge in autonomous driving systems, where improving detection performance typically comes at the cost of increased model complexity, conflicting with the lightweight requirements of edge deployment. To address this dilemma, this paper proposes LEAD-YOLO (Lightweight Efficient Autonomous Driving YOLO), an enhanced network architecture based on YOLOv11n that achieves superior small object detection while maintaining computational efficiency. The proposed framework incorporates three innovative components: First, the Backbone integrates a lightweight Convolutional Gated Transformer (CGF) module, which employs normalized gating mechanisms with residual connections, and a Dilated Feature Fusion (DFF) structure that enables progressive multi-scale context modeling through dilated convolutions. These components synergistically enhance small object perception and environmental context understanding without compromising network efficiency. Second, the neck features a hierarchical feature fusion module (HFFM) that establishes guided feature aggregation paths through hierarchical structuring, facilitating collaborative modeling between local structural information and global semantics for robust multi-scale object detection in complex traffic scenarios. Third, the head implements a shared feature detection head (SFDH) structure, incorporating shared convolution modules for efficient cross-scale feature sharing and detail enhancement branches for improved texture and edge modeling. Extensive experiments validate the effectiveness of LEAD-YOLO: on the nuImages dataset, the method achieves 3.8% and 5.4% improvements in mAP@0.5 and mAP@[0.5:0.95], respectively, while reducing parameters by 24.1%. On the VisDrone2019 dataset, performance gains reach 7.9% and 6.4% for corresponding metrics. These findings demonstrate that LEAD-YOLO achieves an excellent balance between detection accuracy and model efficiency, thereby showcasing substantial potential for applications in autonomous driving.

## 1. Introduction

With the acceleration of global urbanization and the continuous growth of transportation demands, intelligent transportation systems (ITSs) have become a key technological means to improve road safety and optimize resource utilization. As a core component, autonomous driving technology is experiencing rapid development, showing great potential in improving traffic efficiency, reducing accident rates, and enhancing travel experiences. However, the environmental perception system, as the “eyes” of autonomous driving, faces many severe challenges, particularly the problem of small object detection in complex dynamic scenarios. Recent accident investigations, including the 2018 Uber autonomous vehicle fatality, have revealed that failures in detecting pedestrians, cyclists, and road debris—which appear as small objects in perception systems—constitute significant safety risks. In actual road scenes, traffic participants such as pedestrians, bicycles, motorcycles, and distant vehicles often appear as small-scale objects in imaging systems. Especially when located at greater distances, their pixel proportion typically accounts for only 1–2% or even less of the entire image. These distant small objects, due to sparse feature information, blurred texture details, and susceptibility to lighting and weather conditions, are often ignored or misdetected by mainstream detection models. Undetected small objects often become potential safety hazards that may lead to catastrophic consequences, as early detection is crucial for predictive path planning and meeting regulatory safety standards such as Euro NCAP’s AEB tests. Meanwhile, the actual deployment of autonomous driving systems also faces the critical challenge of computational resource limitations. Due to factors such as power consumption, heat dissipation, and cost, vehicle computing platforms have far less computational capability than high-performance servers in data centers. Current high-performance detection models mostly rely on deep network structures and complex computational mechanisms. Although they have achieved breakthrough accuracy on standard datasets, they are difficult to run stably on resource-constrained edge devices. Therefore, ensuring small object detection accuracy while achieving model lightweight (i.e., reducing model parameters and computational complexity while maintaining performance) through network structure optimization, parameter compression, and other means to achieve a balance between accuracy and efficiency has become a key research topic for promoting autonomous driving from research prototypes to large-scale commercial applications.

With the rise of deep learning technology, especially Convolutional Neural Networks (CNNs), deep learning-based object detection algorithms have achieved breakthrough improvements in real-time performance and accuracy [1]. These algorithms automatically learn hierarchical feature representations, significantly outperforming traditional handcrafted features in detection accuracy and generalization ability. Current deep detection algorithms are mainly divided into two categories: two-stage and one-stage approaches. Two-stage algorithms like the R-CNN series [2] adopt a “propose-then-classify” strategy, achieving high detection accuracy, particularly in complex scenes and occluded objects. However, their multi-stage processing flow results in high computational overhead, slow inference speed, and substantial hardware requirements, making them unsuitable for resource-constrained autonomous driving platforms. In contrast, one-stage algorithms such as SSD (Single-Shot MultiBox Detector) [3] and YOLO (You Only Look Once) series [4] provide more efficient solutions by directly performing end-to-end detection on feature maps. These methods transform object detection into a unified regression problem, significantly improving inference speed while maintaining competitive accuracy through multi-scale feature fusion mechanisms, making them more suitable for real-time applications.

It is worth noting that, in recent years, the DETR (Detection Transformer) [5] series algorithms based on Transformer architecture have gained widespread attention in academia for their innovative end-to-end detection framework. DETR uses Transformer’s [6] self-attention mechanism to directly predict object sets, avoiding the complex anchor design and non-maximum suppression (NMS) post-processing in traditional methods. However, despite DETR’s elegant theoretical design, it faces severe challenges in practical applications: slow training convergence, high computational resource requirements, and relatively slow inference speed. These factors severely limit its deployment feasibility on resource-constrained vehicle platforms.

In practical applications of autonomous driving, the industry generally adopts one-stage detection methods, mainly due to their three major advantages: fast inference speed that can meet strict real-time detection requirements; end-to-end training methods that simplify system design and deployment; and with technological development, their detection accuracy has approached or even exceeded two-stage methods. Although the early SSD algorithm performed excellently in speed, it was gradually phased out due to its shortcomings in small object recognition and model scalability. In contrast, the YOLO series has become the mainstream solution for object detection in the autonomous driving field with its excellent speed performance, stable detection accuracy, and good scalability. However, facing the special challenges in autonomous driving scenarios—especially small object detection and lightweight deployment requirements—the YOLO series still has room for improvement. Therefore, how to enhance small object perception capabilities by optimizing network structure while maintaining or even reducing computational complexity has become a core research topic.

In response to the above research status, this paper focuses on the deep optimization of the YOLOv11 model structure, systematically constructing a lightweight and structurally efficient object detection network LEAD-YOLO (Lightweight Efficient Autonomous Driving YOLO) around the two core challenges of small object detection performance and model lightweight. This framework achieves performance breakthroughs through four key technical innovations: the lightweight detail-aware module CGF (Convolutional Gated Transformer) significantly enhances the model’s spatial detail perception ability for small objects; the dilated receptive field fusion structure DFF (dilated feature fusion) effectively extends the model’s perception range of small objects and their surrounding environmental information; at the feature fusion level, the hierarchical feature fusion module HFFM (Hierarchical Feature Fusion Module) further strengthens the representation ability of multi-scale objects; additionally, the lightweight shared detection head SFDH (Shared Feature Detection Head) significantly improves the inference efficiency while ensuring detection accuracy, designed with edge deployment constraints in mind for autonomous driving scenarios.

## 2. Related Work

Although the YOLO series models perform excellently in object detection, many problems remain unsolved in complex road environments. Particularly in autonomous driving scenarios, the problems of insufficient small object detection accuracy and excessive model computational complexity severely restrict their practical applications. Therefore, domestic and foreign scholars have carried out extensive structural optimization and improvement work based on YOLO models, mainly focusing on feature enhancement, network lightweight, and multi-scale fusion directions.

### 2.1. Feature Enhancement and Receptive Field Optimization

To improve YOLO models’ perception capabilities in complex scenes, researchers have explored expanding receptive fields and enhancing feature expression. Wang et al. [7] extended the network’s receptive field while embedding the parameter-free SimAM attention mechanism to enhance feature expression without increasing model complexity. Although this method achieved a small improvement in accuracy, the detection speed hardly improved, with limited overall performance enhancement. Similarly, Li et al. [8] introduced dilated convolutions with different dilation rates in the backbone network to expand receptive fields for better small object detection while maintaining the same parameter count. This method improved small object detection performance on the KITTI dataset, but the computational cost increased considerably.

### 2.2. Lightweight Architecture Design

Addressing the resource limitations of vehicle platforms, lightweight design has become a research focus. Luo et al. [9] proposed the YOLOv8-Ghost-EMA model, fusing the lightweight Ghost module with the dynamically weighted EMA mechanism to improve computational efficiency while enhancing feature extraction capabilities. This method achieved significant parameter and computational reduction, though challenges remained in small object and occluded object detection. Zhang et al. [10] proposed a knowledge distillation-based solution, using large YOLO models as teacher networks to guide lightweight student networks, achieving substantial parameter reduction while maintaining most of the original accuracy on the BDD100K dataset. Chen et al. [11] designed DCNv3-Lite, combining deformable convolution with depthwise separable convolution to maintain adaptability to non-rigid objects like pedestrians and bicycles while significantly reducing the computational complexity compared to YOLOv7.

### 2.3. Multi-Scale Feature Fusion Strategies

The core challenge of small object detection lies in insufficient feature information, making multi-scale feature fusion crucial. Yuan et al. [12] enhanced small object detection by introducing multi-scale feature enhancement modules (MFI) and lightweight attention mechanisms (LM) based on YOLOv11, though lacking validation in multi-category scenarios. Liu et al. [13] proposed an adaptive feature pyramid network (AFPN) that dynamically adjusts fusion strategies by learning feature importance weights at different scales, improving small object detection on the Waymo Open Dataset while reducing computational cost through feature channel pruning. Zhao et al. [14] designed a lightweight BiFPN version, achieving the high-precision detection of distant vehicles and pedestrians on the nuScenes dataset through optimized cross-scale connections and weighted feature fusion.

In summary, existing research has made certain progress in YOLO’s small object detection and lightweight, but still has the following limitations: (1) Most methods focus on single optimization objectives, making it difficult to balance accuracy improvement and model lightweight simultaneously; (2) The insufficient consideration of the specificity of autonomous driving scenarios, lacking adaptability to complex conditions such as extreme weather and lighting changes; (3) lightweight design often comes at the cost of sacrificing small object detection performance, posing safety hazards in practical applications. Therefore, how to design a comprehensive optimization solution that can effectively improve small object detection accuracy while significantly reducing model complexity remains a key problem to be solved.

### 2.4. YOLOv11 Network Introduction

YOLOv11 is the latest generation object detection framework released by Ultralytics on 30 September 2024, offering five model scales: n, s, m, l, and x. This series constructs a multi-level model configuration from lightweight to high-performance by systematically adjusting network depth and width, aiming to adapt to diverse application scenarios from resource-constrained devices to high-computing platforms, providing flexible solutions for vision tasks at different levels.

**Figure 1:**
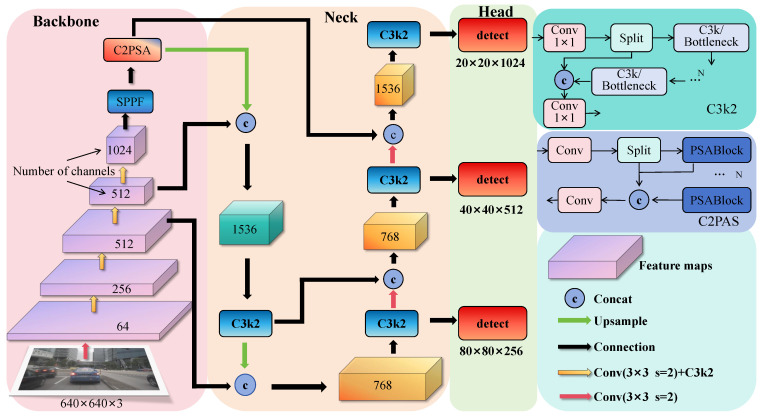
YOLOv11 network structure, the operations represented by different colored arrows have been marked.

The overall architecture of the YOLO11 network consists of three parts: backbone network (backbone), neck network (neck), and detection head (head) (see Figure 1). In the backbone and neck parts, YOLO11 replaces the original C2f module with the C3K2 module, which performs the fine-grained splitting of feature maps through dual-kernel design and bottleneck layers, enhancing both the richness of feature expression and feature extraction speed [15,16]. After the spatial pyramid pooling (SPPF) module, a C2PSA module based on extended C2f is added, introducing a PSA attention mechanism based on multi-head attention and feedforward neural networks (FFNs). This not only strengthens the focusing ability on key features but also optimizes gradient flow through optional residual connections, further enhancing training stability and effectiveness [17,18]. Finally, two depthwise separable convolutions are introduced in the detection head, significantly reducing the computational burden and improving overall operational efficiency [19].

## 3. Proposed Method

The YOLOv11 baseline model still has several issues to be optimized in practical applications. First, the computational cost of its backbone network is relatively high. Although the C3k2 module is introduced to improve the information flow through feature map splitting and small kernel operations, the multi-layer convolution stacking still brings considerable computational burden, which is not conducive to lightweight deployment. Second, the receptive field scales of the original SPPF module are relatively discrete, making it difficult to flexibly interface with different semantic levels, resulting in difficulties in balancing spatial details and global semantic expression when fusing multi-scale features. Additionally, there are still performance bottlenecks in small object detection. Since small objects are often distributed in areas with complex backgrounds and strong interference, while the feature maps generated by the YOLOv11 backbone network have low resolution, it is difficult to effectively capture their detailed features, thereby affecting the detection accuracy.

**Figure 2:**
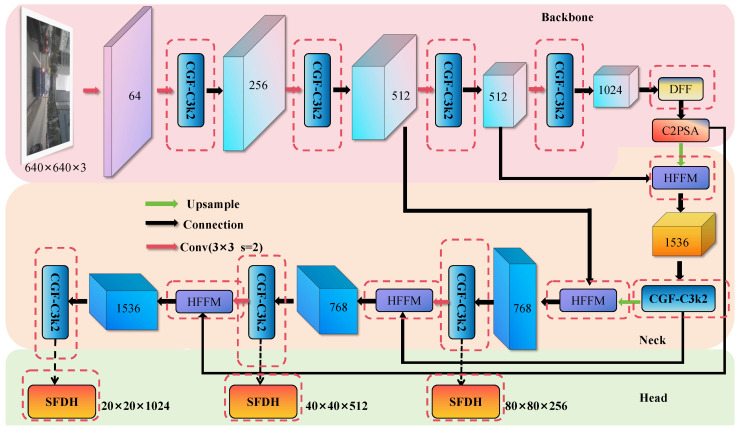
LEAD-YOLO network structure, the red dashed line indicates the improved part.

In view of the above problems, this paper designs and proposes a better-performing detection model LEAD-YOLO based on the lightweight model YOLOv11n. Figure 2 shows the architecture of the LEAD-YOLO model, with red dashed lines indicating the improved parts.

In the backbone, we introduce the lightweight detail-aware module CGF to replace the original bottleneck, enhancing spatial feature extraction capabilities for small objects while maintaining network efficiency and stability of deep information transmission. Addressing the limited modeling capability of the SPPF module, we design the dilated receptive field fusion structure DFF, which uses multi-scale dilated convolutions to achieve progressive context modeling, improving perception of objects and their environment. In the neck part, we propose the hierarchical feature fusion module HFFM, guiding the collaborative modeling of local and global information to enhance detection robustness for multi-scale objects. For the detection head, we construct the lightweight shared structure SFDH, improving cross-scale feature utilization efficiency through shared convolution and detail enhancement branches.

### 3.1. CGF Block Design

The C3k2 structure in YOLOv11’s backbone network faces critical limitations in autonomous driving scenarios. Its fixed convolutional receptive fields struggle to model the spatial dependencies required for detecting small objects in dense traffic, while deep stacking causes feature degradation that impairs fine-grained representations.

Although the Transformer architecture performs well in handling long-range dependencies [20], its high computational cost makes it difficult to be directly applied. The existing gating mechanisms only focus on channel selection, while ignoring the crucial spatial relationships.

**Figure 3:**
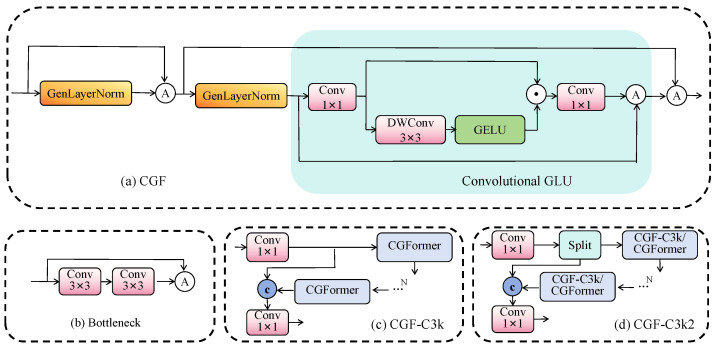
CGF block structure, we use CGF module to optimize the Bottleneck module of C3k2 and C3k.

To address these challenges, we propose the convolutional gated transformer (CGF) module that synergistically combines spatial-aware gating with efficient Transformer-inspired modeling. CGF employs a complementary dual-path design: the gating path preserves fine-grained features against deep network degradation, while the spatial modeling path provides essential neighborhood context. This design is particularly effective for detecting small objects with minimal visual signatures in complex autonomous driving scenarios. The CGF module is strategically designed to optimize the Bottleneck (Figure 3b) structures in C3k (Figure 3c) and C3k2 (Figure 3d) modules, achieving three critical objectives: (1) enhanced spatial sensitivity for small object features, (2) computational efficiency suitable for edge deployment, and (3) stable gradient flow throughout deep networks. Its detailed architecture is illustrated in Figure 3a.

The CGF module implements its dual-path design through two complementary components. For an input feature map $X\in R^{C\times H\times W}$, the transformation follows:

**Spatial modeling path:** To address the fixed receptive field limitation, we first apply dimension permutation and channel-wise normalization:

(1) $$X_{1}=X+{\mathrm{Scale}}_{r}\cdot {\mathrm{Scale}}_{l}\cdot \mathrm{DropPath}\left(\mathrm{LayerNorm}\left(\mathrm{Permute}\left(X\right)\right)\right)$$

This path normalizes features across spatial dimensions to alleviate distribution shift in deep networks. The DropPath mechanism (p = 0.1) provides regularization, while Layer Scale (${\mathrm{Scale}}_{l}=10^{-6}$) prevents training instability.

**Channel gating path:** To prevent feature degradation and enhance fine-grained details, we employ a convolutional GLU (CGLU) mechanism:

(2) $$X_{2}=X_{1}+{\mathrm{Scale}}_{r}\cdot {\mathrm{Scale}}_{l}\cdot \mathrm{DropPath}\left(\mathrm{CGLU}\left(\mathrm{LayerNorm}\left(X_{1}\right)\right)\right)$$

The CGLU splits input features into main branch $F_{m}$ and gating branch *G* through 1 × 1 convolution. The main branch extracts local dependencies via depthwise convolution with GELU activation, while the gating branch dynamically controls feature transmission through element-wise multiplication:

(3) $$\mathrm{CGLU}\left(X\right)=\mathrm{Dropout}({\mathrm{Conv}}_{1\times 1}\left(F_{m}^{\prime }⊙G\right))+X$$

This gating mechanism acts as a learnable filter, selectively amplifying task-relevant features crucial for small object detection while suppressing noise.

The complete CGF transformation $\mathrm{CGF}\left(X\right)=\mathrm{Permute}\left(X_{2}\right)$ achieves synergistic benefits: the spatial path captures neighborhood context essential for understanding object relationships in traffic scenes, while the channel path preserves and enhances fine-grained features through adaptive gating. Multiple residual connections with learnable scaling factors ensure stable gradient flow throughout deep networks. This design maintains computational efficiency through lightweight depthwise convolutions, making it suitable for real-time autonomous driving applications while significantly improving small object detection performance.

### 3.2. DFF Block Design

**Figure 4:**
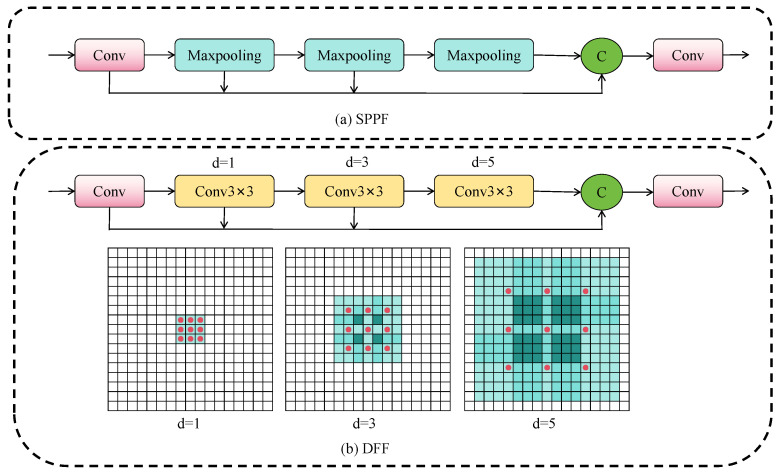
DFF block, d = 1, 3, and 5 represent different dilated convolution rates.

Multi-scale object detection faces challenges in receptive field adaptability and fine-grained feature expression, particularly for distant small objects whose low resolution and weak texture lead to feature loss during downsampling. The conventional SPPF module (Figure 4a) addresses this through multi-scale max pooling but suffers from two limitations: fixed discrete receptive field scales that lack flexibility for different semantic levels, and pooling-induced information compression that damages small object details.

We propose the dilated feature fusion (DFF) module (Figure 4b) to address these limitations. Given input feature $X\in R^{C\times H\times W}$ DFF first applies channel compression through $X_{0}={\mathrm{Conv}}_{1\times 1}\left(X\right)$ to reduce dimensionality by factor r. Subsequently, it constructs a multi-scale feature hierarchy using a shared 3×3 convolution kernel with progressively increasing dilation rates: $X_{i}={\mathrm{Conv}}_{3\times 3}\left(X_{i-1},d=2^{i}\right)$ for i=1,2,…,n, where each layer expands the receptive field while maintaining parameter efficiency. The final output $Y=\mathrm{Concat}[X_{0},X_{1},\ldots ,X_{n}]$ aggregates features across all scales. This recursive structure progressively expands receptive fields from 3×3 to $\left(2^{n+1}+1\right)\times \left(2^{n+1}+1\right)$, preserving fine-grained details crucial for small object detection while capturing multi-scale context through dilated convolutions. Compared to SPPF’s parallel pooling, DFF’s sequential dilation mechanism better models spatial hierarchy without information loss, particularly benefiting distant small object detection where feature preservation is critical.

### 3.3. HFFM Block Design

Feature fusion is a key link in achieving the accurate perception of multi-scale objects and an understanding of environmental semantics, directly affecting the model’s recognition ability for small objects and complex backgrounds in autonomous driving scenarios. However, traditional methods often have difficulty balancing spatial details and global semantic expression when fusing multi-source features from different scales, resulting in incomplete feature information expression and insufficient discriminative ability. Especially when facing small objects with limited fine-grained information, simple concatenation easily introduces redundant background noise and causes key object features to be overwhelmed by large-scale features, thereby reducing model discriminative ability and detection accuracy.

To solve the above problems, we design the Hierarchical Feature Fusion Module HFFM to introduce into the Neck part, aiming to achieve the collaborative modeling of local structure and global semantics by constructing hierarchical feature selection paths.

**Figure 5:**
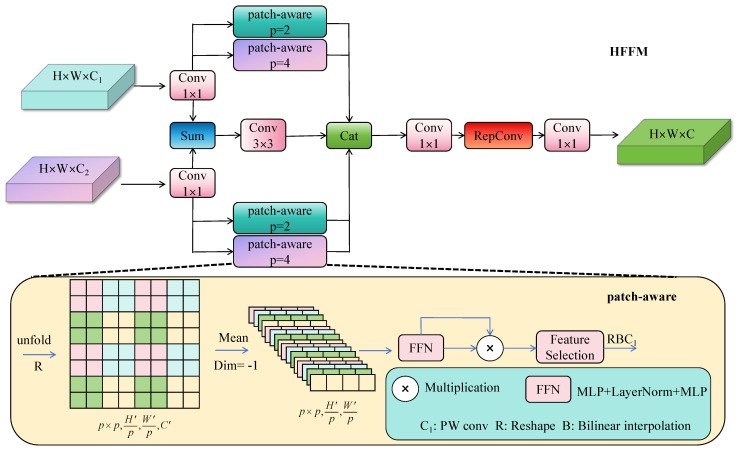
HFFM block: the parameters p of the patch-aware convolution are 2 and 4, which represent the local and global branches, respectively.

Given two input features $F_{1}\in R^{C\times H\times W}$ and $F_{2}\in R^{C\times H\times W}$, HFFM operates in three stages (Figure 5):

Stage 1: Dimension alignment and baseline fusion. First, we align feature dimensions and establish a baseline fusion:

(4) $$F_{i}^{\prime }={\mathrm{Conv}}_{1\times 1}\left(F_{i}\right),\;F_{\mathrm{base}}={\mathrm{GroupConv}}_{3\times 3}\left(\left[F_{1}^{\prime },F_{2}^{\prime }\right]\right)$$

where $F_{i}^{\prime }\in R^{C_{\mathrm{mid}}\times H\times W}$ aligns channel dimensions. This baseline preserves general fusion information while reducing computation through group convolution.

Stage 2: Hierarchical feature extraction. To capture multi-scale patterns, we process aligned features through patch-aware attention [21] with different receptive fields:

(5) $$F_{\mathrm{local}}=\mathrm{PatchAware}\left(F_{1}^{\prime },p=2\right),\;F_{\mathrm{global}}=\mathrm{PatchAware}\left(F_{2}^{\prime },p=4\right)$$

The PatchAware module performs spatial attention within p×p patches:

(6) $$\mathrm{PatchAware}\left(X,p\right)=X⊙\sigma ({\mathrm{AvgPool}}_{p\times p}\left(\mathrm{Conv}\left(X\right)\right))$$

The smaller patch size (p = 2) preserves fine-grained details crucial for small objects, while larger patches (p = 4) capture global context, creating complementary feature representations.

Stage 3: Adaptive feature aggregation. Finally, we aggregate all features and apply learnable selection:

(7) $$\mathrm{HFFM}\left(F_{1},F_{2}\right)={\mathrm{Conv}}_{1\times 1}(\mathrm{RepConv}({\mathrm{Conv}}_{1\times 1}([F_{\mathrm{local}},F_{\mathrm{global}},F_{\mathrm{base}}])))$$

The reparameterizable convolution (RepConv) [22] enables efficient inference while the cascaded convolutions perform channel-wise feature selection, suppressing redundancy and highlighting salient regions.

This hierarchical design ensures that small object features are preserved through dedicated local branches while maintaining necessary global context, effectively addressing the multi-scale detection challenge in autonomous driving scenarios.

### 3.4. SFDH Block Design

Although YOLOv11 performs well in object detection, its detection head suffers from (1) Different scale branches are independent, lacking sharing and collaboration, increasing computational redundancy, which is not conducive to small object detection; (2) limited detail perception for distant objects. We propose the shared feature detail-enhancement head (SFDH) to address these issues through unified multi-scale processing and explicit texture enhancement.

**Figure 6:**
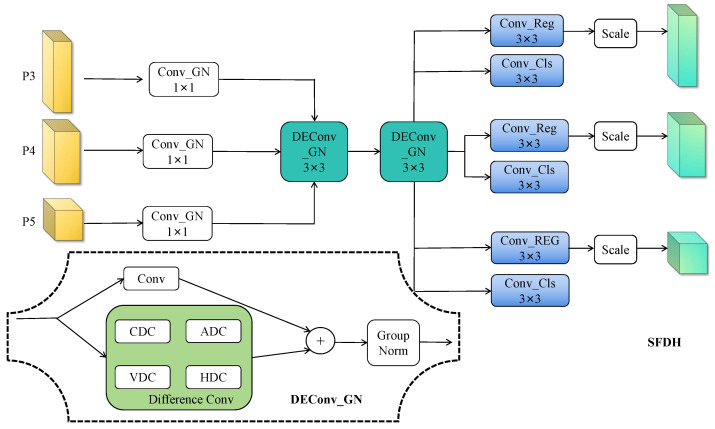
SFDH block, blocks of the same color represent shared convolutions.

As illustrated in Figure 6, SFDH first unifies multi-scale features ${\left\{F_{i}\right\}}_{i=1}^{3}$ through channel projection: $F_{i}^{\prime }={\mathrm{Conv}}_{\mathrm{proj}}\left(F_{i}\right)\in R^{C_{\mathrm{proj}}\times H\times W}$. This provides consistent input dimensions for subsequent shared processing.

The core innovation lies in the shared DEConv modules that process all scales jointly: $F_{i}^{\prime \prime }={\mathrm{DEConv}}_{2}\left({\mathrm{DEConv}}_{1}\left(F_{i}^{\prime }\right)\right)$. Each DEConv module [23] decomposes convolution into five complementary paths capturing different geometric patterns. Specifically, it combines a learnable standard convolution $K_{\mathrm{std}}$ with four fixed difference operators: center difference $K_{CD}$ for texture edges, horizontal $K_{\mathrm{HD}}$, and vertical $K_{\mathrm{VD}}$ for directional structures, and angular $K_{\mathrm{AD}}$ for diagonal patterns. Each path applies group normalization and GELU activation: $F_{\mathrm{path}}=\mathrm{GELU}\left(\mathrm{GN}\left({\mathrm{Conv}}_{\mathrm{path}}\left(X\right)\right)\right)$.

During inference, these multiple paths reparameterize into a single convolution through kernel fusion:

(8) $$K_{\mathrm{fused}}=K_{\mathrm{std}}+\sum_{j\in \{CD,HD,VD,AD\}}K_{j}$$

This design maintains the enhanced feature representation learned during training while reducing inference to a single 3×3 convolution operation.

Finally, shared prediction heads generate outputs with scale-adaptive calibration:

(9) $${\mathrm{reg}}_{i}=s_{i}\cdot {\mathrm{Conv}}_{\mathrm{reg}}\left(E_{i}^{\prime \prime }\right)\times r$$

(10) $${\mathrm{cls}}_{i}={\mathrm{Conv}}_{\mathrm{cls}}\left(E_{i}^{\prime \prime }\right)$$

where learnable scale factors $s_{i}$ compensate for varying object sizes across detection levels and *r* denotes regression granularity.

By sharing detail enhancement modules across all scales and introducing geometric priors through specialized kernels, SFDH reduces parameters by 40% compared to the original head while significantly improving small object detection—a critical requirement for autonomous driving applications.

## 4. Experiments and Results Analysis

### 4.1. Datasets and Data Processing

The nuImages [24] dataset is an image subset of the nuScenes autonomous driving dataset released by Motional, specifically serving image-level vision tasks. This dataset covers typical traffic scenarios such as urban main roads, residential areas, and suburban roads, with collection conditions including various weather (sunny, cloudy, rainy) and lighting environments (daytime, dusk, night), as well as different traffic density conditions, fully reflecting the diversity and complexity of autonomous driving scenarios, making it an ideal dataset for evaluating autonomous driving visual perception capabilities. The dataset contains images captured by six cameras from different angles, totaling 93,000 images covering 25 different category information with rich and high-quality annotation information.

**Figure 7:**
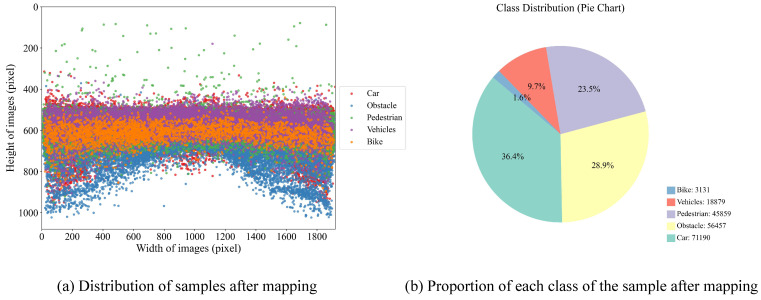
Sample distribution and proportion after mapping.

To meet the needs of forward-view object detection in autonomous driving scenarios, this paper selects 18,368 image samples collected by the vehicle’s front camera. The official division of this portion of data has been completed, including 13,187 training images, 3249 validation images, and 1932 test images. Considering the simplification and generalization requirements for object semantic classification in practical applications, we removed the driveable surface class and integrated the remaining 24 visual object classes into five categories based on semantic attributes: pedestrian, obstacle, bike, car, and vehicles, with specific mapping relationships shown in Table 1. The distribution of mapped object categories is shown in Figure 7a, and the proportion of each category is shown in Figure 7b. Statistical results show that the three categories are dominated by small objects—pedestrian, obstacle, and bike—account for up to 88.8% of the overall samples. This characteristic highlights the representativeness and pertinence of this dataset in small object detection tasks for autonomous driving scenarios, possessing high research value and practical application potential.

VisDrone2019 [25] is a large-scale drone vision dataset released by Northeastern University in China, specifically for object detection and tracking tasks from drone perspectives. The dataset contains over 25,000 images covering diverse scenarios such as cities, rural areas, highways, and construction sites, using various shooting angles including overhead and oblique views, covering different lighting and weather conditions. The dataset annotates common object categories such as pedestrians, vehicles, bicycles, and motorcycles, providing detailed annotation information including bounding boxes, categories, occlusion conditions, and motion states. This study adopts its official standard division, using 6471 training images and 548 validation images. This division follows the dataset’s original settings, ensuring the comparability of experimental results with other research, while the remaining images are used as test sets for final performance evaluation.

### 4.2. Experimental Environment and Parameters

This experiment uses the Autodl computing cloud platform with the Ubuntu 22.04 operating environment, GPU model RTX 3090 (24GB), CPU Intel(R) Xeon(R) Platinum 8358P. The development language is Python 3.10, the deep learning framework is PyTorch 2.2.2, and CUDA version is 11.8. Experimental training parameters are shown in Table 2.

### 4.3. Evaluation Metrics

This experiment uses precision (P), recall (R), mAP@0.5, mAP@[0.5:0.95], computational cost (GFLOPs), and parameters as evaluation metrics to measure detection accuracy and model lightweight effects. The calculations for P, R, AP, and mAP are shown in the following equations:

(11) $$P=\frac{TP}{TP+FP}$$

(12) $$R=\frac{TP}{TP+FN}$$

(13) $$AP=\int_{0}^{1}P\left(R\right)dR$$

(14) $$mAP=\frac{1}{N}\sum_{i=1}^{N}AP_{i}$$

where true positive (TP) represents the number of samples correctly predicted as positive, false positive (FP) represents the number of negative samples incorrectly predicted as positive, false negative (FN) represents the number of positive samples incorrectly predicted as negative. AP (average precision) represents the average precision of a single detection category, $AP_{i}$ is the average precision of the i-th detection category, and mAP is the average precision of all detection categories.

Precision refers to the proportion of actual objects among those predicted as positive by the model. High precision means fewer false positives in the model’s predictions, with a high reliability of detected objects.

Recall refers to the proportion of objects correctly detected by the model among all actual objects. High recall means fewer missed detections, with the model able to discover as many objects as possible.

mAP@0.5 represents the model’s ability to successfully identify objects with a relatively loose overlap criterion (IoU≥0.5) in object detection tasks. It comprehensively considers whether the objects detected by the model are correct and the detection accuracy. Higher mAP@0.5 indicates stronger accuracy in object localization and classification, with fewer missed detections and false positives.

mAP@[0.5:0.95] reflects the model’s average detection performance under various strictness levels (IoU gradually increasing from 0.5 to 0.95). It requires not only good object detection but also more precise detection box positions, reflecting the model’s detailed detection capabilities.

Computational cost refers to the number of floating-point operations required during model inference, used to measure the degree of computational resource consumption. Lower computational cost means fewer computational resources required during model operation, usually achieving a faster inference speed and lower power consumption, making it more suitable for real-time applications or edge device deployment scenarios with resource constraints.

Parameters represent the number of learnable weights in the model, reflecting model complexity and size. Fewer parameters mean smaller space required for model storage and transmission, making it more convenient for deployment on storage-limited devices.

### 4.4. Comparative Experiments

#### 4.4.1. C3k2 Improvement Comparative Experiment

To verify the effectiveness of the proposed CGF module, we also selected other C3k2 module improvement schemes using different internal structures for comparative analysis. All models were trained under consistent training processes to ensure fair comparison of various structural improvement schemes. As shown in Table 3, the CGF-C3k2 module reduced the computational cost from 6.3 GFLOPs to 5.7 GFLOPs while improving mAP, with a 14.7% reduction in parameters. This module achieves a good balance between accuracy and model computational efficiency, demonstrating high optimization potential.

#### 4.4.2. Neck Improvement Comparative Experiment

Similarly, to evaluate the effectiveness of the proposed neck improvement structure, we also selected several typical schemes for comparative experiments. As shown in Table 4, although the computational cost slightly increased by 0.9 GFLOPs and parameters increased by 3.5%, the model achieved significant performance improvements. Specifically, mAP@0.5 improved by 2.3% and mAP@[0.5:0.95] improved by 2.5%, indicating that this improvement effectively enhanced the model’s detection capabilities. With limited computational resource growth, the improved module achieved significant accuracy improvements.

#### 4.4.3. Head Improvement Comparative Experiment

To verify the effectiveness of the detection head improvement, different detection heads were trained under consistent training processes. The results shown in Table 5 indicate that the SFDH detection head improved mAP@0.5 by 1.5% while reducing computational cost by 0.2 GFLOPs and parameters by 13.4%, demonstrating strong performance improvement and resource utilization efficiency.

#### 4.4.4. Model Comparison Before and After Training

To more clearly demonstrate the changes in detection accuracy, recall, and average precision for different categories, we trained the original YOLOv11n (A) model and LEAD-YOLO (B) model on the nuImages dataset while maintaining consistent hyperparameters and training settings. Table 6 provides detailed comparison data before and after improvement. From Table 6, it can be seen that LEAD-YOLO’s detection performance improvement is particularly significant for small object categories such as pedestrian, obstacle, and bike, with mAP@0.5 improving by 3.7, 2.7, and 3.4 percentage points, respectively, and mAP@[0.5:0.95] improving by 3.8, 5.4, and 4 percentage points, respectively. This indicates that the proposed method has more advantages in small object recognition and localization, verifying the enhancement effect of the improved structure on fine-grained object perception capabilities.

#### 4.4.5. Different Model Comparative Experiment

To further verify the improvement and performance benefits, we compare LEAD-YOLO with two main categories of mainstream algorithms: one is a one-stage detector, and the other is a two-stage detector and a Transformer based variant of DETR. The one-stage categories include SSD, YOLOv5s [34], YOLOv7-tiny [35], YOLOv8n [36], YOLOv11, and other popular small object detection methods include EfficientDet-D0 [37] and RetinaNet [38]. The two-stage detector and a Transformer based variant of DETR includes Faster R-CNN, DETR-R18, and Def-DETR-R50(Deformable DETR-R50). All experiments used identical training parameters.

As shown in Table 7, LEAD-YOLO demonstrates significant advantages when compared with mainstream one-stage detectors. In terms of detection performance, LEAD-YOLO achieves the best results across all evaluation metrics, with a precision (P) of 74.6%, recall (R) of 56.6%, mAP@0.5 of 64.2%, and mAP@[0.5:0.95] of 35.2%. Compared to the second-best performing YOLOv11n, LEAD-YOLO improves mAP@0.5 by 3.8 percentage points and mAP@[0.5:0.95] by 2.4 percentage points. Moreover, LEAD-YOLO achieves optimal computational efficiency with only 6.1 GFLOPs and 1.928 million parameters. Compared to YOLOv11n, it reduces the computational cost by 3.2% and parameters by 26.3%. When compared with traditional detectors like SSD and RetinaNet, the parameter reduction reaches 92.3% and 94.3%, respectively, fully demonstrating the advantages of the lightweight design.

Table 8 presents the comparison results with two-stage detectors and Transformer-based DETR variants. While faster R-CNN maintains leading detection accuracy (mAP@0.5: 68.7%, mAP@[0.5:0.95]: 37.3%), it comes with extremely high computational cost, requiring 206.2 GFLOPs and 41.39 million parameters. In contrast, LEAD-YOLO achieves a 97.0% reduction in computational cost and 95.3% reduction in parameters with only a 4.5% performance gap in mAP@0.5, demonstrating an excellent efficiency–accuracy balance. Furthermore, LEAD-YOLO slightly outperforms both DETR-R18 and Def-DETR-R50 in detection performance while maintaining overwhelming advantages in computational efficiency. Compared to DETR-R18, it reduces the computational cost by 87.3% and parameters by 92.6%, proving that CNN-based lightweight designs still hold significant advantages in small object detection tasks.

**Figure 8:**
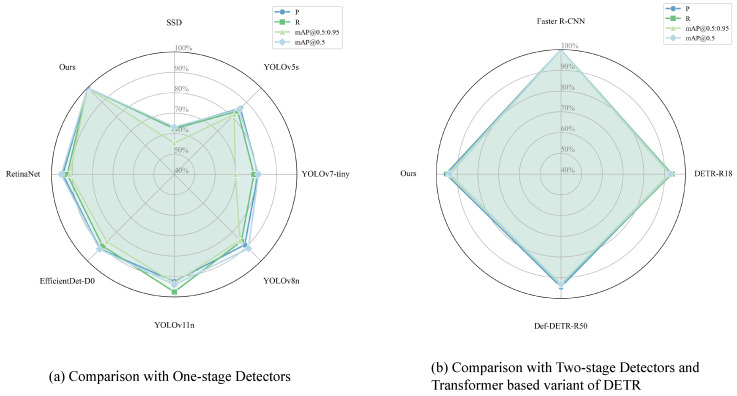
Comparative experimental performance analysis.

The performance analysis illustrated in Figure 8 further validates the comprehensive advantages of LEAD-YOLO. Experimental results demonstrate that LEAD-YOLO successfully achieves the design goal of maintaining competitive detection accuracy while significantly reducing model complexity. This efficient performance is primarily attributed to (1) carefully designed feature extraction and fusion mechanisms; (2) detection head structures optimized for small objects; and (3) effective model compression strategies. These characteristics suggest LEAD-YOLO’s potential for resource-constrained edge computing environments in real-time small object detection tasks.

#### 4.4.6. Generalization Performance Evaluation Experiment

To verify the generalization performance and robustness of our proposed improvement method, we conducted cross-dataset experiments on the VisDrone2019 dataset. We selected YOLOv5s, YOLOv8n, YOLOv11n, and RTDETR-R18 as comparison algorithms. Experimental results are shown in Table 9.

Experimental results show that LEAD-YOLO demonstrates superior detection performance on datasets with more small objects. Compared to YOLOv11n, its mAP@0.5 improved by 7.9%. Meanwhile, LEAD-YOLO maintains the lowest model parameters while achieving the highest detection accuracy. In cross-dataset generalization testing, this method still maintains good detection effects, verifying its effectiveness and robustness in small object detection tasks.

### 4.5. Ablation Experiments

#### 4.5.1. DFF Module Ablation Experiment

The DFF module concatenates three weight-shared convolution modules with different dilation coefficients. To verify the impact of different dilation coefficient configurations in multi-scale feature modeling, we compared the performance of common [1, 2, 3], [1, 2, 6], [1, 3, 9] and our [1, 3, 5] three groups of dilation rate settings in the DFF module. Experimental results are shown in Table 10.

**Figure 9:**
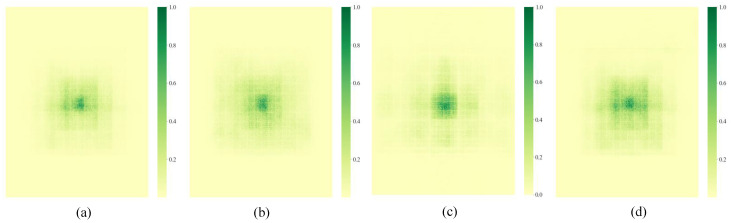
Receptive fields at different dilation rates.

From the table, it can be seen that, when the dilation rates are [1, 3, 5], both mAP@0.5 and mAP@[0.5:0.95] achieve the best results. Figure 9 shows the actual receptive field distribution maps corresponding to the four groups of dilation rates. It can be seen that [1, 2, 3] (Figure 9a) has limited receptive field expansion, making it difficult to effectively cover long-distance context information. At the same time, due to adjacent dilation rates, multiple convolution operations will repeatedly act on the same set of elements, resulting in a large amount of redundant computation in local areas with low information fusion efficiency. Some areas in [1, 2, 6] (Figure 9b) still repeatedly convolved due to the large dilation rate span, causing certain resource waste, and the central area has high weights. In [1, 3, 9] (Figure 9d), the receptive field expands but there is no information exchange between the convolution operators of the second and third layers, which will result in the inability to obtain more complete feature information.

The [1, 3, 5] configuration designed in this paper (Figure 9c) not only expands the receptive field size but also has smoother step changes between layers, making the middle layer features (second layer) more balanced in obtaining context information, with stronger structural symmetry and computational balance, helping to improve the coherence and fusion ability of multi-scale features. Additionally, this configuration establishes more balanced information paths between edge and central regions, reducing the occurrence of areas not effectively covered by convolution. Experiments also show that integrating DFF into the detection backbone significantly improves the detection accuracy and robustness of distant objects.

#### 4.5.2. Overall Ablation Experiment

**Figure 10:**
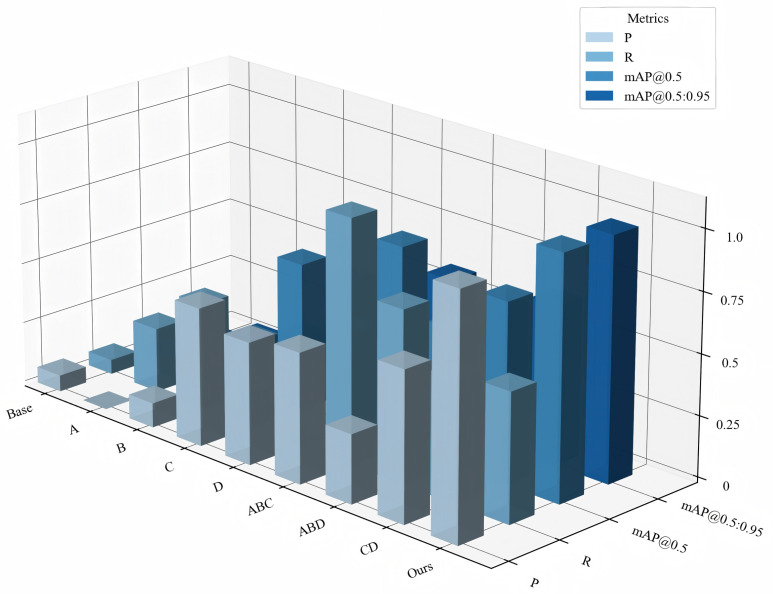
Analysis of ablation experiments.

To verify the effectiveness of the core module designs proposed in this paper, we designed and implemented a series of ablation experiments on the nuImage dataset. In all ablation experiments, YOLOv11n was used as the baseline model, with key modules gradually introduced or replaced to analyze the impact of each component on overall performance. The CGF and DFF modules form a complete ’detail-context’ perception system in the Backbone. CGF focuses on protecting detail features of small objects from deep network degradation through gating mechanisms, while DFF is responsible for expanding the receptive field to capture the contextual environment of these detail features. This design concept requires the two modules to work together: the detail features preserved by CGF need the context information provided by DFF for accurate localization, while the expanded receptive field of DFF needs the high-quality features maintained by CGF to avoid semantic ambiguity. Therefore, conducting ablation experiments on them as a whole better reflects their design intent. Table 11 shows the experimental results under various ablation configurations, with the result analysis shown in Figure 10. From the figure, it can be seen that each improvement enhances the model’s detection performance to varying degrees. After introducing the lightweight module CGF, while improving mAP by 0.4%, computational cost was reduced to the lowest 5.7 GFLOPs. After combining CGF with DFF and HFFM, the model recall rate reached the highest value of 58.2%. Finally, the model integrating all improved modules achieved maximum gains of 3.8% and 5.4% in mAP@0.5 and mAP@[0.5:0.95], respectively, while reducing the computational cost by 0.2 GFLOPs and parameters by 24.1%. Overall, the improved model achieves a comprehensive improvement in detection accuracy based on reducing parameters and computational complexity, especially showing outstanding performance in small object detection, demonstrating significant advantages in object detection tasks in actual autonomous driving scenarios.

### 4.6. Visualization Analysis

#### 4.6.1. Detection Comparison

**Figure 11:**
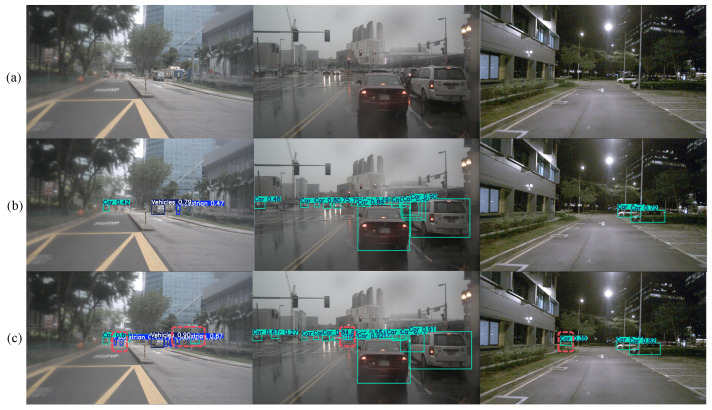
Detection comparison in various scenarios: The red dotted box in the figure is the part where the improved algorithm has better detection effect than the baseline algorithm.

This experiment uses YOLOv11n and its improved model LEAD-YOLO for testing and comparison on the test set. Figure 11a shows images under sunny, rainy, and night scenes, respectively, Figure 11b shows YOLOv11n model detection results, and Figure 11c shows LEAD-YOLO model detection results. The red dashed boxes in the figures indicate parts where the improved algorithm performs better than the baseline algorithm. Through comparison, it can be seen that the improved algorithm can more accurately detect distant pedestrians and other small objects in sunny environments; successfully identifies distant obstacles in rainy and partially occluded scenes; and can accurately detect distant vehicles under strong light interference at night. The proposed improved model demonstrates better detection capabilities in various complex scenarios, further verifying the effectiveness and robustness of the improved model in practical applications.

#### 4.6.2. Heatmap Comparison

To further analyze the contribution of the proposed improved structures to model performance improvement, we use gradient-based visualization methods to display the heatmaps of intermediate feature responses for different models.

**Figure 12:**
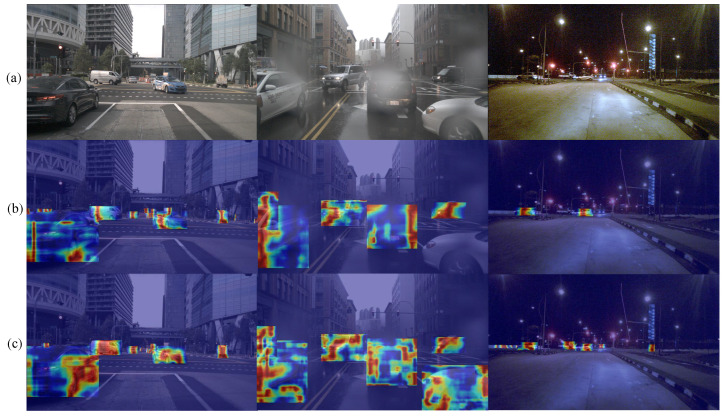
Detection comparison in various scenarios: The red dotted box in the figure is the part where the improved algorithm has better detection effect than the baseline algorithm.

Figure 12a shows the images of different road environments under three typical weather conditions: sunny, rainy, and night. Figure 12b,c, respectively, correspond to the heatmap visualization results of the baseline model and improved model in the above scenarios, where red areas indicate regions with stronger model attention.

From the heatmaps, it can be observed that the baseline model has certain limitations in object detection tasks, with its attention regions prone to shift and insufficiently clear responses at object boundaries, especially showing unstable performance in scenarios with dense distant small objects or partial occlusion. The model with introduced improvement modules can effectively enhance response capabilities for key object regions, with activation regions more concentrated on object edge contours and semantically significant structural regions, while suppressing the redundant activation of background areas. This optimization of attention mechanisms not only improves the model’s feature expression capabilities but also enhances the object discrimination, further verifying the actual effects of structural improvements in detection accuracy improvement.

## 5. Conclusions

In autonomous driving scenarios, small object detection has always been a key challenge affecting model practicality and safety. Especially in edge device deployment with limited computational resources, detection models face higher requirements for lightweight and high accuracy. Existing detection models cannot solve the above problems well, so we propose the improved lightweight detection model LEAD-YOLO. First, in the backbone part, we design the lightweight detail-aware module CGF to optimize the bottleneck part in the C3k2 module, achieving lightweight design while maintaining stable flow of deep semantic features. Then, we design the DFF module to replace the original SPPF module. Compared to the SPPF module, DFF focuses more on the progressive modeling of multi-scale context, enhancing the perception capabilities for semantic relationships between small objects and complex backgrounds through dilated convolution. In the neck part, we propose the HFFM module to construct multi-level feature fusion paths, achieving efficient interaction between local details and global context through hierarchical perception mechanisms, improving the model’s adaptability to multi-scale objects in complex traffic scenarios. For the detection head, we design the lightweight shared structure SFDH, achieving efficient cross-scale feature fusion through shared convolution modules and introducing detail enhancement branches focusing on local edge and texture modeling, significantly reducing model complexity while improving object recognition capabilities, suitable for real-time deployment scenarios in autonomous driving.

Experimental results on the nuImages dataset show that LEAD-YOLO improves mAP@0.5 by 3.8% and mAP@[0.5:0.95] by 5.4% compared to the baseline model while maintaining extremely low computational overhead, with a 24.1% reduction in parameters, fully demonstrating its good balance performance between lightweight and accuracy. On the VisDrone2019 dataset, LEAD-YOLO also shows excellent performance, with mAP@0.5 and mAP@[0.5:0.95] improving by 7.9% and 6.4%, respectively, compared to the baseline, further verifying the robustness and practicality of this method in complex scenarios. Moreover, LEAD-YOLO’s detection performance improvement on small object categories (such as pedestrian, obstacle, and bike) in the nuImages dataset is particularly significant: mAP@0.5 improved by 3.7%, 2.7%, and 3.4%, respectively, and mAP@[0.5:0.95] improved by 3.8%, 5.4%, and 4.0%, respectively, indicating that the proposed structure has stronger capabilities in fine-grained object perception and localization, providing a more advantageous solution for small object detection tasks in autonomous driving systems.

Future work will explore three main directions to enhance the model’s capabilities in autonomous driving scenarios:

1. Adaptive network structure design: We will develop dynamic architectures that can adjust model complexity based on real-time scenario analysis. This involves implementing scene-aware gating mechanisms that automatically select appropriate network configurations—using lighter branches for simple highway scenes while activating deeper feature extraction paths for complex urban intersections. This adaptive approach could reduce the computational overhead by 30–50% in simple scenarios while maintaining full capacity for challenging conditions.

2. Environmental robustness enhancement: We will design specialized modules targeting extreme conditions, including (i) weather-adaptive attention mechanisms that dynamically recalibrate features based on detected weather patterns (fog, rain, snow), (ii) illumination-invariant feature extraction using learnable histogram equalization for robust day/night performance, and (iii) context-aware processing that adjusts detection parameters based on environmental factors.

3. Multi-modal fusion and temporal modeling: Integration of temporal information through recurrent connections will enable the tracking of object motion patterns, improving small object detection through temporal consistency. Additionally, fusion with LiDAR or radar data will provide complementary depth information, which is particularly beneficial for distant small object detection.

These advancements will be validated on diverse datasets including BDD100K, Waymo Open Dataset, and the extreme weather subsets of nuScenes, ensuring robust performance across varied autonomous driving conditions.

## Figures and Tables

**Table 1:** Dataset sample class mapping relationship.

Original Categories	Mapped Category
Adult, child, pedestrian	Pedestrian
Bicycle rack, construction cone,	Obstacle
Debris, pushable pullable object,	
Traffic cone, barrier	
Bicycle, motorcycle	Bike
Personal mobility, car,	Car
emergency vehicle, police car	
Bus, construction vehicle,	Vehicles
trailer, truck, ambulance,	
bendy bus, rigid bus	

**Table 2:** Experimental training parameters.

Parameter	Value
Image size (imgs)	640×640
Initial learning rate (lr0)	0.01
Optimizer	SGD
Batch size	16
Epochs	300
Momentum	0.937
Weight decay	0.0005

**Table 3:** C3k2 improvement comparison experiment.

Model	P(%)	R(%)	mAP@0.5(%)	mAP@[0.5:0.95](%)	GFLOPs	Params
Base	**69.0**	55.0	60.4	32.8	6.3	2,616,248
C3k2-Faster [26]	68.0	50.8	57.5	30.2	5.9	2,322,096
C3k2-FAT [27]	66.2	52.0	58.2	31.0	6.7	2,654,188
C3k2-JDPM [28]	67.5	53.2	59.6	31.6	7.9	2,954,477
CGF-C3k2	68.6	55.7	60.8	33.2	5.7	2,231,836

**Table 4:** Neck improvement comparison experiment.

Model	P(%)	R(%)	mAP@0.5(%)	mAP@[0.5:0.95](%)	GFLOPs	Parameters
Base	69.0	55.0	60.4	32.8	6.3	2,616,248
MFM [29]	68.4	54.3	59.5	31.3	6.9	2,633,464
ASF [30]	71.2	55.0	61.8	33.6	6.7	2,163,976
Hyper [31]	70.5	54.8	61.3	34.2	7.7	3,064,888
HFFM	72.0	55.3	62.7	35.3	7.2	2,707,128

**Table 5:** Detection head improvement comparison experiment.

Model	P(%)	R(%)	mAP@0.5(%)	mAP@[0.5:0.95](%)	GFLOPs	Parameters
Base	69.0	55.0	60.4	32.8	6.3	2,616,248
Dyhead [32]	68.4	54.2	59.8	31.4	7.6	3,133,108
PGI [33]	71.3	54.9	61.7	33.5	8.8	3,604,864
SFDH	71.6	54.8	61.9	34.6	6.1	2,265,435

**Table 6:** Performance comparison of five categories between YOLOv11n(A) and LEAD-YOLO(B).

Categories	P(%)		R(%)		mAP@0.5(%)		mAP@[0.5:0.95](%)	
	A	B	A	B	A	B	A	B
Pedestrian	69.2	74.8	47.6	47.3	53.4	57.1	24.7	28.5
Obstacle	69.9	77.4	60.6	59.6	63.9	66.6	31.9	37.3
Bike	59.3	69.0	48.6	47.6	50.4	53.8	25.7	29.7
Car	75.9	79.2	67.9	71.4	75.3	78.9	46.9	53.5
Vehicles	70.1	72.5	50.3	57.2	58.8	64.6	34.9	42.2
All	69.0	74.6	55.0	56.6	60.4	64.2	32.8	38.2

**Table 7:** Comparison with one-stage detectors.

Model	P(%)	R(%)	mAP@0.5(%)	mAP@[0.5:0.95](%)	GFLOPs	Parameters
SSD	47.0	35.3	40.6	19.5	31.4	25,082,528
YOLOv5s	64.0	47.5	54.6	28.7	15.9	7,039,792
YOLOv7-tiny	60.4	44.7	52.1	24.6	9.5	6,034,656
YOLOv8n	66.2	48.9	58.6	30.1	8.1	3,426,452
YOLOv11n	69.0	55.2	60.4	32.8	6.3	2,616,248
EfficientDet-D0	68.3	50.7	58.9	30.5	13.0	3,915,671
RetinaNet	70.5	52.3	61.2	32.1	115.8	34,052,412
Ours	74.6	56.6	64.2	35.2	6.1	1,927,696

**Table 8:** Comparison with two-stage detectors and Transformer-based variant of DETR.

Model	P(%)	R(%)	mAP@0.5(%)	mAP@[0.5:0.95](%)	GFLOPs	Parameters
Faster R-CNN	78.2	60.1	68.7	37.3	206.2	41,393,461
DETR-R18	72.8	56.2	63.8	34.4	48.2	26,158,465
Def-DETR-R50	73.9	56.1	64.0	34.7	52.7	45,546,846
Ours	74.6	56.6	64.2	35.2	6.1	1,927,696

**Table 9:** Comparative experiments on different datasets.

Model	P(%)	R(%)	mAP@0.5(%)	mAP@[0.5:0.95](%)
YOLOv5s	42.7	32.8	31.2	18.3
YOLOv8n	37.3	29.4	26.2	15.6
YOLOv11n	36.5	28.6	25.8	14.2
RTDETR-R18	40.1	30.6	28.6	17.3
Ours	45.2	34.7	33.7	20.6

**Table 10:** Module effect under different dilation coefficients.

Model	mAP@0.5(%)	mAP@[0.5:0.95](%)
Base	60.4	32.8
1,2,3	59.3	32.3
1,2,6	60.8	33.1
1,3,5	61.2	33.6
1,3,9	60.6	32.9

**Table 11:** Ablation experiment.

Model	CGF	DFF	HFFM	SFDH	P(%)	R(%)	mAP@0.5(%)	mAP@[0.5:0.95](%)	GFLOPs	Parameters
Base					69.0	55.0	60.4	32.8	6.3	2,616,248
A	✓				68.6	55.7	60.8	33.2	5.7	2,231,836
B		✓			69.2	56.3	61.2	33.6	6.5	2,763,704
C			✓		72.0	55.3	62.7	35.3	7.2	2,707,128
D				✓	71.6	54.8	61.9	34.6	6.1	2,265,435
ABC	✓	✓	✓		71.8	58.2	63.5	36.1	6.3	2,129,272
ABD	✓	✓		✓	70.3	57.2	62.5	35.8	6.1	2,412,891
CD			✓	✓	72.3	56.5	63.2	36.3	6.5	1,967,275
ABCD(Ours)	✓	✓	✓	✓	74.6	56.6	64.2	38.2	6.1	1,927,696

## Data Availability

The data that support the findings of this study are available from the author, Y.Y., upon reasonable request.
